# The Not-So-Dark Side of Materialism: Can Public Versus Private Contexts Make Materialists Less Eco-Unfriendly?

**DOI:** 10.3389/fpsyg.2019.00790

**Published:** 2019-04-05

**Authors:** Luxiao Wang, Dian Gu, Jiang Jiang, Ying Sun

**Affiliations:** Beijing Key Laboratory of Applied Experimental Psychology, National Demonstration Center for Experimental Psychology Education (Beijing Normal University), Faculty of Psychology, Beijing Normal University, Beijing, China

**Keywords:** materialism, pro-environmental behaviors, public contexts, private contexts, impression management theory

## Abstract

Materialism, a way of life characterized by pursuing possessions, image, and status, has always been looked upon as self-interested and unkind. Previous studies have widely verified that materialism has a negative impact on individuals’ pro-environmental behaviors. The present research focused on whether the public (versus private) nature of a decision context will make materialists behave in more eco-friendly ways. In Study 1, the behavioral decision context (public vs. private) was manipulated to examine whether the relationship between materialism and pro-environmental behaviors would vary as a function of the situation. In Study 2, we manipulated materialism and contexts simultaneously to verify the hypothesis again. Findings in the two studies consistently revealed that public versus private contexts played a moderating role between materialism and pro-environmental behaviors. That is, in private, individuals with higher levels of materialism were less eco-friendly than those with lower levels of materialism, but the negative effect disappeared in public. We concluded with a discussion of the theoretical and practical implications of the research findings.

## Introduction

With economic development, individuals are increasingly pursuing the ownership of material wealth and economic success. This growing, prevalent value is described as materialism by researchers (Richins, 2004; Kasser, 2016). Materialism is “a psychological construct reflecting the extent to which an individual believes that it is important to attain money, possessions, image, and status, relative to other aims in life” (Kasser, 2018). It has been widely documented that materialism is detrimental for individuals in many aspects of life (for a review, see Kasser, 2016), such as self-esteem (Chaplin and John, 2007; Nagpaul and Pang, 2017), well-being (Dittmar et al., 2014; Jiang et al., 2016; Wang et al., 2017), financial and consumption behaviors (Donnelly et al., 2012; Dittmar et al., 2014), interpersonal relationships (Kasser and Ryan, 2001), and ecological attitudes and behaviors (Hurst et al., 2013). Meanwhile, the increasingly serious environmental problems have prompted researchers’ concern regarding how to promote humans’ beneficial coexistence with the environment. Therefore, considering the negative effect of materialism on pro-environmental behaviors, it is worth noting how to motivate materialists to be environmentally friendly. In the present study, we aimed to explore the moderating role of public versus private contexts between materialism and pro-environmental behaviors based on the impression management theory.

Pro-environmental behaviors refer to behaviors that consciously seek to reduce the negative effect of individuals’ actions on the environment (Kollmuss and Agyeman, 2002). The negative effect of materialism on pro-environmental behaviors has been widely demonstrated both theoretically and empirically. Theoretically speaking, according to Schwartz’s value model (Schwartz, 1992; Schwartz et al., 2012), individuals who place a relatively higher priority on self-enhancement value (e.g., materialism) are less likely to regard self-transcendent value (e.g., environmentalism) as important. In other words, materialism is in conflict with environmentalism, which is the value behind pro-environmental behaviors. When individuals hold materialistic values, they will care little about environmentalism, and thus, they will be less likely to display pro-environmental behaviors. Consistent with this theory, empirical studies have revealed the negative effect of materialism on pro-environmental behaviors. A meta-analysis reported that materialism had a medium and stable negative effect on pro-environmental behaviors across 15 studies (Hurst et al., 2013), which indicates that individuals with higher levels of materialism are more likely to adopt lifestyles with a high ecological footprint. For example, materialists are less likely to classify and recycle household waste or reuse plastic bags and bottles (Ku and Zaroff, 2014) and tend to consume more energy (Witt et al., 2014; Andersson and Nässén, 2016) in their daily lives. Apart from these tendencies, materialists are reluctant to donate money (Ku and Zaroff, 2014) or join environmental organizations (Kilbourne and Pickett, 2008) to improve the environment. Moreover, the negative impact of materialism on the environment has also been found at the regional level. Previous studies have found that the more a region prioritized materialistic values, the more energy was consumed (Gu et al., in press) and the more CO_2_ was emitted (Kasser, 2011). More importantly, the focal studies mainly concerned pro-environmental behaviors in private contexts and failed to control decision contexts. Although many studies have focused on the negative effects of materialism on the environment, no studies so far have provided empirical evidence on how to mitigate the focal negative impact.

In fact, individuals’ behaviors are affected not only by personal values but also by certain situational factors (Magnusson and Stattin, 1998). That is, the negative effect of materialism on pro-environmental behaviors may vary in different situations. In the present study, we mainly focused on decision contexts to explore if the public versus private nature of the context can moderate associations between materialism and pro-environmental behaviors. According to the impression management theory, people are motivated to shape a positive public image (Schlenker, 1985). Some previous studies have revealed that exposure to public contexts made individuals behave more prosocially (Haley and Fessler, 2005; Linardi and Mcconnell, 2012), such as being more honest (Shu et al., 2012) and more generous (Leimgruber et al., 2012). However, a recent meta-analysis found that there was no evidence to support that artificial cues of being watched increase generosity (Northover et al., 2016). Regarding the inconsistent results, Pfattheicher and Keller (2015) suggested the inconsistent results based on the impression management theory in previous studies may result from different people holding different levels of impression management motives. That is to say, individuals would be affected by public contexts only when they were sensitive to the evaluation of others.

Previous studies have used the impression management theory to explore the interaction effect of the public versus private nature of context and certain individual factors on prosocial behaviors, such as Machiavellianism (Bereczkei et al., 2010; Bereczkei and Czibor, 2014), narcissism (Ding et al., 2016) and personal socioeconomic status (Kraus and Callaghan, 2016). The results consistently showed that individuals with higher levels of Machiavellianism, narcissism, and socioeconomic status tended to behave more altruistically in public contexts, but in private contexts, they would not “pretend to be good” and instead showed fewer prosocial behaviors. And importantly, materialists have some traits in common with Machiavellianism and narcissism (Mchoskey, 1999). For example, both materialists and Machiavellianism believe it is important to attain financial success, and in common with narcissism, materialists attach importance to striving for appealing image. Thus, it is reasonable to speculate that public (versus private) contexts could also offer an opportunity to motivate materialists’ pro-environmental behaviors.

Pro-environmental behaviors, as a kind of prosocial behavior (Ramus and Killmer, 2010), can provide individuals with a prosocial reputation (Lin-Healy and Small, 2013). For example, it has been shown that individuals tend to overreport their pro-environmental behaviors to cater to social norms as a result of social desirability bias (Barr, 2007; Kormos and Gifford, 2014). Indeed, prosocial reputation is important for human beings (Rand and Nowak, 2013). As previous studies have revealed, prosocial reputation could help individuals obtain more money (Hardy and van Vugt, 2006), enhance their interpersonal attractiveness (Barclay, 2004), and improve their social status (Price, 2003). And such reputational concerns are more salient in public contexts according to the impression management theory and previous studies (Flynn et al., 2006; Dan and Baumard, 2012).

For materialists, some evidence suggests that the prosocial reputation is particularly important. On one hand, individuals with higher levels of materialism are more likely to manage their public images. For example, Dermody et al. (2015) believed that Chinese materialists would want to participate in green consumption because of impression management motivation. Moreover, individuals with higher levels of materialism attach more importance to evaluations of themselves from the public (Wong, 1997; Xu, 2008). That is to say, compared to nonmaterialists, materialists attach more importance to impression management motives and, thus, are more sensitive to evaluations from the public. On the other hand, the benefits of prosocial reputation are also in accordance with the construct of materialism. That is, individuals with higher levels of materialism attach more importance on possessions, appealing image, and high status. Therefore, individuals with higher levels of materialism will behave in more eco-unfriendly ways in private contexts, because the value that motivates pro-environmental behaviors is in conflict with materialistic values. However, they may display fewer eco-unfriendly behaviors to obtain a prosocial reputation in public contexts, because the reputation and its subsequent benefits are compatible with their goals.

Aligned with the discussion above, we hypothesized that the public versus private nature of decision context would moderate the negative effect of materialism on pro-environmental behaviors. Materialists will behave in less eco-unfriendly ways in public contexts. Specifically, materialism negatively associated with pro-environmental behaviors in private contexts, and the negative association disappeared in public contexts. In the present study, we conducted two experimental studies to verify our hypothesis. Study 1 preliminarily explored whether the association between materialism and pro-environmental behaviors would vary in different contexts (public vs. private). In Study 2, we tried to verify the result again and clarify the causal effect of interaction between materialism and context on pro-environmental behaviors by manipulating the two factors simultaneously. Apart from this, regarding the measurement of pro-environmental behaviors, it has been noticed that self-reported data was mainly used to assess pro-environmental behaviors in previous related studies (Gu et al., in press). It is difficult to draw accurate conclusions from self-reported data as a result of social desirability bias (e.g., Kormos and Gifford, 2014), consistency bias, participants’ failing to recall behaviors accurately, and so on (Gifford, 2014). Thus, in the present study, a laboratory task—the resource dilemma task (Sheldon and Mcgregor, 2000) was employed to assess pro-environmental behaviors, in which the individual interest of maximizing personal gains conflicts with the collective interest of long-term preservation of the forest resource. The task has also been adapted for the Chinese population (Ku and Zaroff, 2014).

## Study 1

In Study 1, we tested our moderation hypothesis by manipulating whether or not the context-related behaviors were described as public versus private. We proposed that materialism should have a negative impact on pro-environmental behaviors when the behavioral decision is private, yet the negative effect should be eliminated when the behavioral decision is public.

### Method

#### Participants

For our moderation hypothesis, we estimated that a sample size of 103 would be required in order to have 80% power (*α* = 0.05) to detect a medium-sized effect using G*Power 3.1 (Faul et al., 2009). However, considering there was no *via*ble effect size estimate of interaction between materialism and the decision context in prior studies, we recruited as many participants as possible before any data analysis, and none were recruited after the data analysis. More than 100 participants were allotted to each cell of the design, which exceeded the recommendation of 20 observations per cell (Simmons et al., 2011). A total of 224 participants were recruited via an online survey platform (www.wjx.cn) in exchange for monetary compensation, and 42.86% of them were males (*n* = 96). The average age was 33.77 ± 7.30 years (ranging from 19 to 60 years), and the average annual income was ¥179,500 ± 102,200 Yuan.

#### Materials

##### Materialism

The extrinsic motivation subscale in the Aspiration Index (AI; Kasser and Ryan, 1996; Grouzet et al., 2005) one of the most used measurements of materialism and has been previously used in the Chinese population (Kasser, 2016), was used. The extrinsic motivation subscale includes three kinds of material goals: financial success (e.g., possession of wealth), fame (e.g., be famous), and image (e.g., have a stylish haircut and clothing), and each goal is measured by five items. Participants were asked to rate the importance of each item on a 7-point Likert scale ranging from 1 = *unimportant* to 7 = *important*. We computed a mean score for each participant, with higher scores indicating higher levels of materialism. The Cronbach’s *α* coefficients for whole scale, financial success, fame, and image were 0.92, 0.86, 0.82, and 0.85, respectively.

##### Pro-environmental Behaviors

The resource dilemma task (Sheldon and Mcgregor, 2000) was adapted to measure participants’ pro-environmental behaviors. Participants were first asked to imagine that they are the owner of a timber company and must compete with three other companies to harvest timber in the same forest. They need to cut down as many trees as possible for their companies to profit and thrive. However, the rapid deforestation could lead to forest destruction. Then, participants were asked to answer the following questions: “How fast do you want your company to cut down trees?” on a 7-point Likert scale ranging from 1 = *very slow* to 7 = *very fast*, and “How many acres of trees do you expect your company to cut down?” (range from 1 to 100 acres).

#### Procedure

Participants in the present study were randomly assigned to the public condition (*n* = 117) or the private condition (*n* = 107). First, they were asked to complete the assessment of materialism and personal information (i.e., age, gender, annual income). Then, participants in both conditions read the instructions for the resource dilemma task. To manipulate the decision contexts, participants in the public condition had to read extra information: “According to local politics and public opinion, every decision you make should be published on your company’s website and your official WeChat account to ensure that local residents can follow the process of the forest resource utilization in a timely manner,” while participants in the private condition did not need to read additional information. Finally, all the participants answered the two questions about timber harvesting to represent their pro-environmental behaviors. Upon completion, they were thanked and debriefed. All procedures were reviewed and approved by the ethics board of Beijing Normal University.

### Results

First, we conducted an independent sample *t* test to compare the level of materialism between the two conditions. The results showed that there were no differences between the public condition (*M* = 4.95, *SD* = 0.85) and the private condition (*M* = 4.93, *SD* = 1.00), *t* = 0.15, *p* = 0.881, which means that participants in the two conditions were homogeneous in materialism.

**Figure 1:**
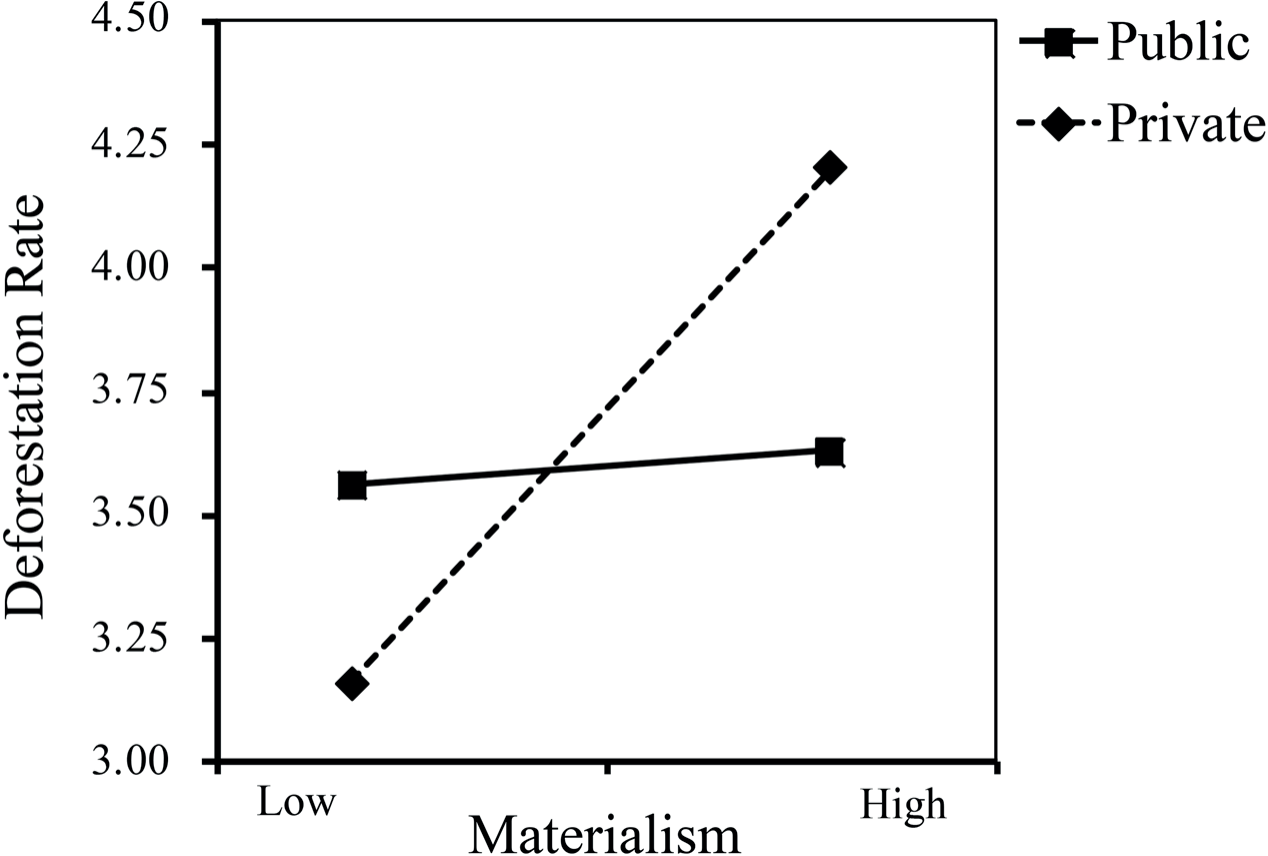
Relationship between materialism and deforestation rate as a function of decision contexts (public vs. private) in Study 1.

Subsequently, to test for the moderating effect of public versus private contexts on the association between materialism and the deforestation rate, we regressed the deforestation rate on materialism, decision contexts (dummy coded: 0 = private, 1 = public) and their interaction by employing the PROCESS macro (Model 1, 5,000 bootstrap samples) for SPSS (Hayes, 2013). The results showed a significant main effect of materialism on the deforestation rate (*B* = 0.47, *SE* = 0.16, *t* = 3.05, *p* < 0.001, 95%CI = [0.16, 0.78]), such that participants with higher levels of materialism were likely to cut trees faster. There was no main effect of decision context on the deforestation rate (*B* = −0.11, *SE* = 0.21, *t* = −0.54, *p* > 0.05, 95%CI = [−0.53, 0.30]). Moreover, a significant materialism × context interaction emerged (*B* = −0.50, *SE* = 0.23, *t* = −2.14, *p* = 0.033, 95%CI = [−0.96, −0.04], Δ*R*^2^ = 0.020), which means that the effect of materialism on deforestation rate was moderated by the public versus private contexts. Specifically, materialism positively predicted deforestation rate in the private condition (*M* = 3.69, *SD* = 1.56; *B* = 0.47, *SE* = 0.16, *t =* 3.05, *p* < 0.001, 95%CI = [0.17, 78], Δ*R*^2^ = 0.091), while in the public condition, materialism had no significant effect on deforestation rate (*M* = 3.58, *SD* = 1.66; *B* = −0.03, *SE* = 0.17, *t =* −0.15, *p* > 0.05, 95%CI = [−0.37, 0.32]) (see Figure 1). Moreover, the results did not change significantly after controlling for gender, age, and annual income. In other words, materialists were more likely to cut trees faster in the private condition, while in the public condition, where their choice could be noticed by the public, they were not likely to cut trees faster.

**Figure 2:**
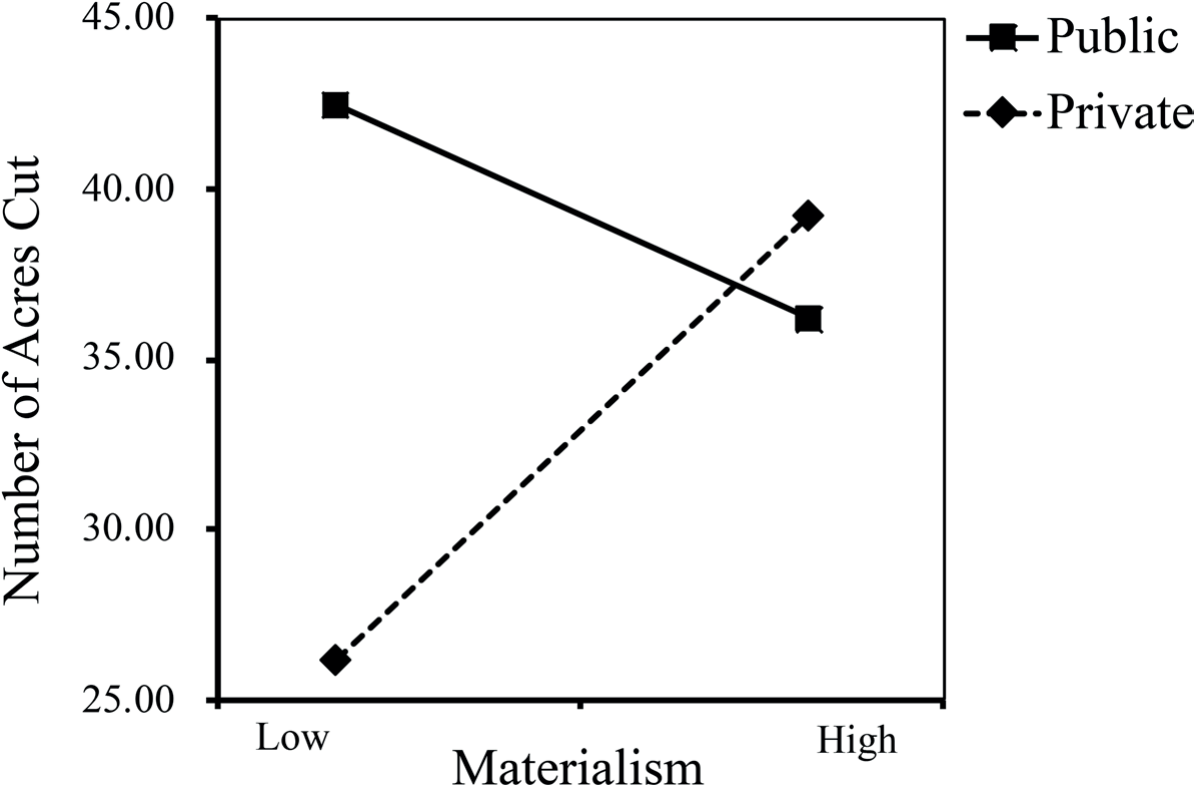
Relationship between materialism and number of acres cut as a function of decision contexts (public vs. private) in Study 1.

Similar to deforestation rate, we conducted a moderation effect analysis to test if public versus private contexts moderated the association between materialism and the number of acres individuals chose to cut. The main effect of materialism on the number of acres participants chose to cut was marginally significant (*B* = 5.11, *SE* = 2.95, *t* = 1.73, *p* = 0.085, 95%CI = [−0.71, 10.92]), and there was no main effect of decision context on the number of acres (*B* = 6.33, *SE* = 4.04, *t* = 1.56, *p* > 0.05, 95%CI = [−1.64, 14.30]). Moreover, the results showed that the moderating effect was marginally significant, *B* = −8.01, *SE* = 4.42, *t* = −1.81, 95%CI = [−16.72, 0.70], *p* = 0.071, Δ*R*^2^ = 0.015. Specifically, the effect of materialism on the number of acres participants chose to cut was positive but marginally significant in the private condition (*M* = 32.85, *SD* = 27.09), *B* = 5.11, *SE* = 2.95, 95%CI = [−0.71, 10.92], *t =* 1.73, *p* = 0.085, Δ*R*^2^ = 0.027, while in the public condition, materialism had no significant effect on the number of acres (*M* = 39.20, *SD* = 33.05), *B* = −2.90, *SE* = 3.29, 95%CI = [−9.29, 3.58], *t =* −0.88, *p* = 0.379 (see Figure 2). Similarly, the results did not change significantly after controlling for demographic variables. The above findings suggested that materialists were likely to cut more trees in the private condition, whereas when their choice could be noticed by the public, they were not likely to cut more trees.

### Brief Discussion

The results of Study 1 provided preliminary evidence that public versus private contexts could moderate the effect of materialism on pro-environmental behavioral intentions. In the private condition, materialists were selfish and more concerned about their own interest than environmental benefits; hence, they were likely to cut trees faster and to cut more trees. However, when participants’ behavior was exposed to public scrutiny, the negative impact of materialism on pro-environmental behavior was improved. That is, public contexts make materialists less eco-unfriendly, which verified our hypothesis. In Study 2, we further manipulated materialism and decision contexts simultaneously to verify the moderating effect again.

## Study 2

In Study 2, we sought to replicate the findings of Study 1 by manipulating the levels of materialism and whether or not context-related behaviors were described as public versus private. Furthermore, the decision contexts were manipulated in different ways from Study 1 to improve the reliability of the results.

### Method

#### Participants

The manipulation of materialism had an unknown main effect size and reflected a somewhat different comparison (high level versus low level of materialism) than the regressions reported in Study 1 (level of materialism to which one hold). We based our decision to determine sample size on a power analysis (through G*Power 3.1, Faul et al., 2009) that assumed we wanted to be able to achieve a statistical power of 80% to detect a medium effect size (*η*^2^ = 0.06) for our moderation hypotheses. This analysis suggested a required sample size of 125 participants. A total of 133 participants (30 males) were recruited from a large university in exchange for ¥15 Yuan. The average age was 22.03 ± 2.72 years (ranging from 17 to 34 years), and three participants failed to report their age. Furthermore, the average monthly consumption was ¥1652.26 ± 823.95 Yuan.

#### Materials

##### Materialism

To test whether participants were homogeneous among different conditions in the baseline level of materialism, participants’ levels of materialism were measured before the manipulation of materialism. The measurement was the same as in Study 1. In the present study, the Cronbach’s *α* coefficient was 0.90.

##### Manipulation of Materialism

We employed the manipulation paradigm from Solberg et al.’s study (2004). Participants were asked to write about the topic of extrinsic or intrinsic goals to manipulate the level of materialism. In the materialism priming condition, participants were required to read the definition of extrinsic goals and then to describe their extrinsic goals, using three keywords to summarize them. In contrast, participants in the control group were required to read the definition of intrinsic goals and then to describe their intrinsic goals, using three key words to summarize them.

A pilot study was conducted to test the effectiveness of the materialism manipulation prior to Study 2. A total of 223 participants (95 males) were recruited. The average age was 30.78 ± 7.48 years (ranging from 15 to 60 years). To check the manipulation effect, participants were asked to complete a State Materialism scale after the manipulation task. This scale was adapted from the original 3-item Material Values Scale (MVS; Richins, 2004), such that items referred to current state of mind (e.g., “At the moment, I admire people who own expensive homes, cars, and clothes”). Each item was rated on a 5-point Likert scale ranging from 1 (*strongly disagree*) to 5 (*strongly agree*). The Cronbach’s *α* coefficient for the scale was 0.75. The results of the manipulation check showed that participants who wrote extrinsic goals (*n =* 107, *M* = 3.46, *SD* = 0.86) had significantly higher scores on state materialism than those who wrote intrinsic goals (*n =* 116, *M* = 3.22, *SD* = 0.94), *t* = 1.98, *p* < 0.05, Cohen’s *d* = 0.27. Such findings indicated that the manipulation of materialism was successful.

##### Pro-environmental Behaviors

As in Study 1, the resource dilemma task was used in the present study. Again, the two questions about timber harvesting were used to measure pro-environmental behaviors in the task.

#### Procedure

Participants were randomly assigned to conditions in a 2 (materialism: prime, control) × 2 (decision context: public, private) between-subjects factorial design. Participants completed an extrinsic motivation subscale in AI and personal information (i.e., age, gender and monthly consumption) approximately 2 days before the formal experiment.

**Figure 3:**
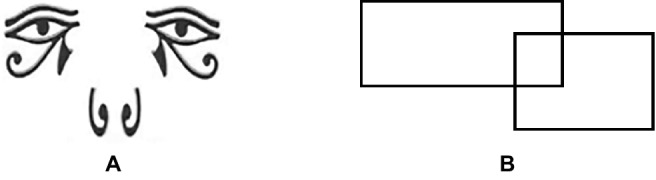
Watermarks used with public **(A)** and private **(B)** groups in Study 2.

In the formal experiment, participants were first asked to write about extrinsic or intrinsic goals to manipulate their levels of materialism. Then, all the participants finished the resource dilemma task on paper questionnaires. During the task, two approaches were used to manipulate decision contexts. Specifically, in the public condition, participants completed the task with the presence of an experimenter, while participants in the private condition completed the task alone (Lee and Wagner, 2002). Moreover, the watermarks on the paper questionnaires differed between the two conditions. The watermark in the public condition was an image of an eye (see Figure 3A), but the watermark in the private condition was an irrelevant figure (see Figure 3B; Haley and Fessler, 2005).

Upon completion, participants were thanked and debriefed. All procedures were reviewed and approved by the ethics board of Beijing Normal University.

### Results

First, we conducted an *F* test to examine whether the samples were homogeneous among the four groups at the baseline level of materialism (materialism-prime and public condition: *n* = 39, *M* = 4.47, *SD* = 0.86; materialism-prime and private condition: *n* = 34, *M* = 4.75, *SD* = 0.81; materialism-control and public condition: *n* = 30, *M* = 4.63, *SD* = 0.91; materialism-control and private condition: *n* = 30, *M* = 4.64, *SD* = 0.79). The results showed that the main effects of materialism, public versus private contexts, and their interaction were not significant, *F*s < 1, *p*s > 0.1, indicating the homogeneity of baseline-level materialism in the four groups.

**Figure 4:**
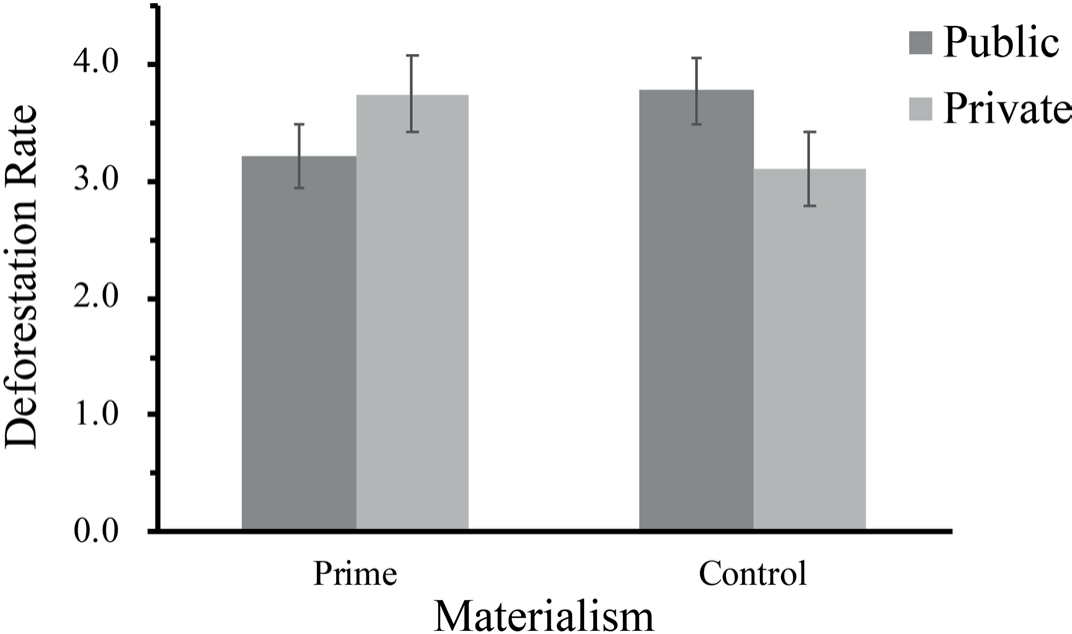
Deforestation rate as a function of materialism and decision contexts (public vs. private) in Study 2.

Subsequently, to examine the interaction effect between materialism and decision contexts on the deforestation rate, a two-factor MANOVA was conducted. The results revealed that the main effect of materialism (*F*(1,129) = 0.05, *p* > 0.05) and decision context (*F*(1,129) = 0.15, *p* > 0.05) was not significant. Furthermore, as expected, the interaction effect between materialism and decision context was significant, *F*(1,129) = 5.64, *p* < 0.05, 95%CI = [−2.31, −0.36], $\eta_{p}^{2}$ = 0.042. Then, simple effect analysis revealed that in the private condition, participants in the materialism priming group (*M* = 3.74, *SD* = 1.64) were likely to cut trees faster than the control group did (*M* = 3.10, *SD* = 1.32), and the result was marginally significant, *F*(1,129) = 3.08, *p* = 0.08, $\eta_{p}^{2}$ = 0.023. However, in the public condition, there is no significant difference between materialism priming group (*M* = 3.21, *SD* = 1.45) and the control group (*M* = 3.77, *SD* = 1.34), *F*(1,129) = 2.56, *p* = 0.11, $\eta_{p}^{2}$ = 0.019. Moreover, after controlling demographic variables, the main effect of materialism and decision context remained non-significant, and the interaction effect was still significant, *F* (1,123) = 8.20, *p* = 0.005, $\eta_{p}^{2}$ = 0.062. Specifically, in the private condition, participants in the materialism priming group were likely to cut trees significantly faster than the control group did, *F*(1,123) = 5.12, *p* < 0.05, $\eta_{p}^{2}$ = 0.040. In contrast, in the public condition, participants in the materialism priming group were likely to cut trees at a slower rate than the control group did, and the result was marginally significant, *F*(1,123) = 3.26, *p* = 0.074, $\eta_{p}^{2}$ = 0.026 (see Figure 4).

**Figure 5:**
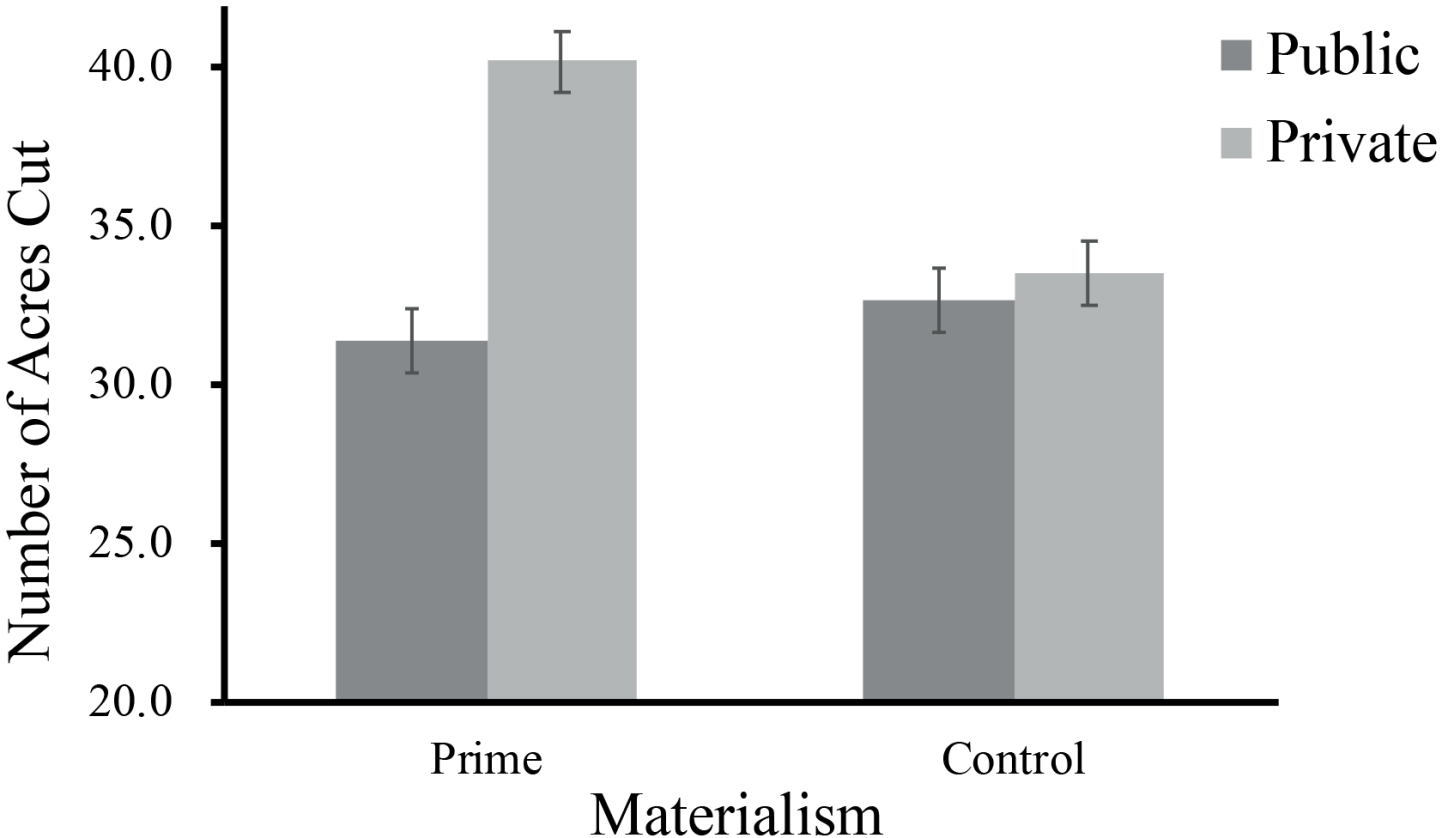
Number of acres cut as a function of materialism and decision contexts (public vs. private) in Study 2.

We also conducted a two-factor MANOVA to examine the interaction effect for the number of acres that individuals chose to cut. Two participants were excluded from the analysis because they failed to answer the question as required. We found that the main effect of materialism (*F*(1,127) = 0.49, *p* > 0.05) and decision context (*F*(1,127) = 1.65, *p* > 0.05) was not significant, nor was the interaction effect, *F*(1,127) = 1.11, *p* = 0.29, 95%CI = [−23.93, 7.28]. Specifically, in the private condition, participants in the materialism priming group (*M* = 40.21, *SD* = 24.82) had a tendency to cut down more trees than the control group did (*M* = 33.60, *SD* = 21.47), *F*(1,127) = 1.49, *p* = 0.23. However, in the public condition, participants in materialism priming group (*M* = 31.40, *SD* = 19.11) and the control group (*M* = 32.73, *SD* = 20.38) showed similar choices in the number of acres they wished to cut, *F*(1,127) = 0.07, *p* = 0.80. And the results did not change significantly after controlling for demographic variables. Although the interaction effect is not statistically significant, the descriptive statistics provided some tentative support for the moderating effect of public versus private contexts (see Figure 5).

### Brief Discussion

In line with the findings obtained in Study 1, the findings in Study 2 verified the moderation hypothesis again by manipulating materialism and decision contexts simultaneously. Specifically, similar to the results in Study 1, we found that individuals with higher levels of materialism tended to cut trees faster than those with lower levels of materialism in the private condition, which means that materialists were likely to behave in eco-unfriendly ways when their behaviors would not be noticed by others. In addition, we found that the negative effect of materialism on pro-environmental behaviors could be reversed to some extent in public contexts (i.e., an image of a watching eye and a real person). When pro-environmental behavioral decisions were noticed and monitored, materialists had a tendency to cut trees at a slower rate. Furthermore, the findings from the number of acres that individuals chose to cut tentatively supported the hypothesis and suggested that the public (vs. private) decision contexts could make materialists choose to cut down fewer trees, which did not confirm the results in Study 1 completely. It is likely that the non-significant results statistically result from the young student identity of the participants in Study 2, who may have no idea what 100 acres of trees actually represent, and then showed biased choices.

## General Discussion

The present research provided evidence to support our hypothesis that public versus private contexts could moderate the negative effect of materialism on pro-environmental behaviors. In public contexts, materialists would behave in less eco-unfriendly or even more eco-friendly ways. Specifically, the results of Study 1 showed that materialists tended to cut trees faster and harvest more forest resources. However, when they believed that their choices would be published on public websites, they would pretend to be good and instead chose to cut fewer trees and to cut more slowly. In Study 2, the causal effect was verified by manipulating materialism and decision contexts simultaneously. We found that individuals who were primed by materialistic values were likely to cut trees faster than those who were primed by nonmaterialistic values in private contexts. Furthermore, the focal effect seemed reversed in public contexts, where one’s behavioral decision could be noticed by an image of a watching eye and experimenter. Above all, these two studies converged to show that exposure to public contexts could make materialists less eco-unfriendly.

Findings about the negative effect of materialism on pro-environmental behaviors are consistent with those of previous studies conducted at the individual and regional levels (Hurst et al., 2013). We found that materialists desired to gain more profit for themselves by plundering more forest resources without considering environmental consequences (i.e., in Study 1), especially when they realized that their behavioral decisions would not be noticed by others (i.e., in Study 2). Importantly, such results also support Schwartz’s value model (Schwartz, 1992; Schwartz et al., 2012). Schwartz (1992) proposed the Circumplex Model and suggested that personal values could form a circular structure. Each value would be similar to, and opposite to, other values, like the self-enhancement value and the self-transcendent value, which are the ends of a “seesaw” (Grouzet et al., 2005). Individuals who have high levels of self-enhancement value often have correspondingly low levels of self-transcendence value. Therefore, materialism, located in the cluster of self-enhancement value, is in conflict with environmentalism, which is located in the cluster of self-transcendence value. Furthermore, individuals with high levels of materialism are often accompanied by low levels of environmentalism, resulting in less eco-friendly behavior.

Moreover, the present study also showed that the negative impact of materialism on the environment could vary in different contexts (public vs. private). To our knowledge, it is the first study to provide empirical evidence about how to mitigate the negative association between materialism and pro-environmental behaviors. Previous studies have found that when social pressure is greater, individuals’ behaviors are less likely to be consistent with their life goals and values (Eom et al., 2016; Unanue et al., 2016). When materialists’ behavioral decisions are being watched by others, these individuals are faced with great social pressure. To some extent, what they do in public will influence how others view them and will also influence their reputations. Hence, materialists seek to conform to social norms and behave in less eco-unfriendly ways to enhance their prosocial reputation, even though such behaviors are not in accordance with their values. In other words, these differences in behavior appear to arise from reputational concerns. Together, the present study highlighted the complex motivations that elicit pro-environmental behaviors and the importance of context in determining the direction of association between materialism and eco-friendliness. More importantly, verification of the moderation hypothesis also implied that the effect of materialism is not always negative. Indeed, numerous studies have found that materialism has a stable, negative impact on personal life and society as a whole (for a review, see Kasser, 2016). However, as shown in the present study, the negative effect of materialism depends on the situation. Drawing on the impression management theory (Schlenker, 1985), the pursuit of prosocial reputation can motivate materialists to shape their behaviors in public contexts.

The findings of the present research have both theoretical and practical implications. Theoretically, the research revealed that the relationship between materialism and pro-environmental behaviors varies as a function of public versus private condition. Materialists behaved better when they were noticed by the public, which is in accordance with the impression management theory (Schlenker, 1985). Individuals think highly of what others think of them, especially in the public condition. More importantly, the present study also provides some initial evidence to explain the inconsistent results about the positive effect of public exposure as mentioned before based on the impression management theory. Consistent with a recent meta-analysis (Northover et al., 2016), we found that there was no main effect of public versus private context in the present study. Similarly, Kraus and Callaghan (2016) explored the moderating effect of decision contexts on associations between social class and prosocial behaviors, and they consistently found there was no main effect of decision contexts across two studies. As the present study revealed, exposure to public contexts could make individuals behave in more eco-friendly ways only for materialists, who are sensitive to prosocial reputation and its subsequent benefits, as previously discussed. Compared to nonmaterialists, materialists may attach more importance to impression management motives and may thus be more sensitive to evaluations from the public. Thus, different people may hold different levels of impression management motives, and then may be affected differently by the public exposure.

Moreover, the results from the present research also provide important practical implications for promoting materialists’ pro-environmental behaviors. Based on the results obtained, we suggest that exposing behavioral decisions to public scrutiny is an effective way to make materialists less eco-unfriendly and, perhaps, even more eco-friendly. For example, the government could consider publicly rewarding residents who use the least energy, which would be beneficial for materialists who hold reputational concerns. In addition to public rewards, adding images of watching eyes to environmental publicity materials (e.g., posters, leaflets) may also foster materialists to behave in more environmentally friendly ways. Moreover, environmental protection agencies can conduct more public environmental protection activities, such as donations and signing up for environmental protection. Individuals with higher levels of materialism would likely participate in such activities in public contexts to obtain prosocial reputation.

There are also some limitations to this research. First, in the present study, we only used a resource dilemma task to measure pro-environmental behaviors, which cannot represent all types of pro-environmental behaviors, especially for common behaviors in daily life, such as reusing and recycling. Thus, future research should measure more types of pro-environmental behaviors to verify the moderation hypothesis, and objective behaviors should be considered in future studies. Previous studies have found that attitudes, behavioral intentions, self-reported behaviors and objective behaviors are not completely consistent (Lammers et al., 2010; Kormos and Gifford, 2014). Second, given that the samples used in the present study were all Chinese, we are limited in the ability to generalize results to different cultures. Indeed, previous studies have shown that in collectivistic cultures such as China, social factors can more strongly predict behaviors rather than personal attitudes, personality, and values (Eom et al., 2016). Thus, future research should use samples from different cultures to retest the moderating effect of public versus private contexts on the negative relationship between materialism and pro-environmental behaviors. Third, it may be difficult to know conclusively what effects (extrinsic goals vs. intrinsic goals) drove the observed results in Study 2, because there was no control group that is neutral in regard to materialism. Thus, we suggest future studies add a neutral group to verify our results. Finally, we advocate that future research shed light on the underlying mechanisms driving the links between materialism × decision contexts and pro-environmental behaviors. As discussed before, pursuing reputation may be the important factor in the focal relationship. Researchers could consider examining whether materialists hold higher levels of reputational concern in public (vs. private) contexts and then choose to behave in less eco-unfriendly ways. We believe that practitioners will be inspired by such research findings to propose interventions to improve the quality of the environment.

## Ethics Statement

This study was carried out in accordance with the recommendations of the Human Protection and the Academic Ethics Committee of Faculty of Psychology at the Beijing Normal University with written informed consent from all subjects. All subjects gave written informed consent in accordance with the Declaration of Helsinki. The protocol was approved by the Academic Ethics Committee of Faculty of Psychology at the Beijing Normal University.

## Author Contributions

LW, YS, and JJ designed the study. LW collected the data. LW and DG analyzed the data. LW and DG drafted the article. DG and JJ provided critical revisions. All authors approved the final version of the article for submission.

### Conflict of Interest Statement

The authors declare that the research was conducted in the absence of any commercial or financial relationships that could be construed as a potential conflict of interest.
